# Veterinary use of bacteriophage therapy in intensively-reared livestock

**DOI:** 10.1186/s12985-019-1260-3

**Published:** 2019-12-12

**Authors:** Adriano Gigante, Robert J Atterbury

**Affiliations:** School of Veterinary Medicine and Science, University of Nottingham, Sutton Bonington, Leicestershire, LE12 5RD UK

**Keywords:** Bacteriophage, Zoonotic, Phage therapy

## Abstract

Zoonoses are infectious diseases transmitted directly or indirectly between animals and humans. Several important zoonotic pathogens colonize farm animals asymptomatically, which may lead to contamination of the food chain and public health hazards. Moreover, routine sampling of carcasses at retail by government authorities over the past 20 years suggests the prevalence of antibiotic resistance in foodborne pathogens has increased. If this continues, antibiotics may be ineffective against such pathogens in the future and alternative approaches, such as phage therapy, may be necessary. Intensive livestock farming is the only realistic way of meeting the demand for meat from an increasing global population and growth in middle class consumers in developing countries, particularly in Asia. This review elaborates on the use of phages to control zoonotic pathogens in intensively-reared livestock (poultry and pigs).

## Background

### Poultry and pig intensive farming

A significant increase in meat production will be required over the coming decades in order to meet demand from a growing global population and greater income and dietary choice in developing countries [1]. So far, much of this demand has been met by intensive livestock farming, particularly poultry and pigs. Unfortunately, such production systems can facilitate disease transmission as these animals often have low genetic diversity and are reared in large and dense populations [2]. The Food and Agricultural Organization (FAO) reported that between 1961 and 2016, world poultry meat production increased from 9 to 120 million tonnes, and egg production grew from 15 to 81 million tonnes [3]. The most recent FAO meat market review report estimated that poultry and pig meat production was 123.9 and 120.5 million tonnes respectively in 2018 [4]. In many parts of the world, antimicrobials are used in intensive farming for growth promotion, disease prevention, or therapy which may select for populations of antibiotic-resistant pathogens [2]. In the USA in 2012, animals consumed 70% of medically important antibiotics (8.9 tons) [5]. In China, the livestock industry will use up to 30% of global antimicrobial production by 2030 [6]. The emergence of antimicrobial resistance in bacterial pathogens will inevitably result in treatment failure, increased pathogen transmission and concomitant production losses [7].

### Bacterial zoonoses and intensively-reared livestock

The most common zoonotic bacterial pathogens associated with poultry and pigs are *Salmonella* spp., *E. coli*, *Campylobacter* spp., *Clostridium* spp. and *Listeria* spp. [8, 9]. The most recent European Food Safety Authority (EFSA) report states that these pathogens are often resistant to several antibiotics [9, 10]. In the EU, the official data about zoonotic and indicator bacteria from humans, animals and food show high proportions (28.6% out of more than 8000) of human *Salmonella* isolates were resistant to three or more antimicrobials [10]. Additionally, 34.9% of indicator isolates of *E. coli* from fattening pigs were multidrug resistant [10]. The pipeline for developing new antibiotics to counter this resistance is running dangerously low on new candidate molecules [11] and alternative approaches are urgently needed. One option is the use of lytic bacteriophages to combat bacterial diseases in livestock [12]. A review sponsored by the UK Department of Health and the Wellcome Trust reported that, of the top 10 most promising alternatives to antibiotics, three were based on using bacteriophages or their components [13].

Bacteriophages were discovered in the early twentieth century by Twort (1915) and d’Herelle (1917) while working independently in the UK and France, respectively [14]. D’Herelle [15] first tested phage therapy in animals, with the successful treatment of fowl typhoid in chickens (95–100% survival of phage-treated birds compared with 0–25% for untreated controls). Pyle [16] reported using phage to treat chickens with a systemic *Salmonella* infection caused by *Salmonella enterica* serotype Pullorum. While the phage demonstrated marked bacteriolysis in vitro; when used in vivo they did not reduce mortality or have much therapeutic effect. Following the discovery of antibiotics in the 1920s, little work was done in the West using phage to treat infections of livestock until Williams Smith’s pioneering studies of the 1980s. For a more extensive review of the history of phage use in agriculture and animals, see the review by Sulakvelidze and Barrow [17]. The following sections summarise findings from more recent phage therapy studies in poultry and pigs.

## Main text

### Salmonellosis

*Salmonella* is a common target for phage therapy because it causes disease in a wide range of endothermic animals as well as humans and causes significant production losses in livestock. Some *Salmonella* serotypes (e.g. *S. enterica* serotype Typhi) are known as ‘host-restricted’ because they produce a severe, systemic, typhoid-like illness in a single host (or small number of related hosts). However, phage therapy has mainly focussed on ‘non-host-restricted’ serotypes (principally Enteritidis and Typhimurium) which usually result in a less severe gastrointestinal infection across a much broader range of species and lead to most foodborne bacterial infections in developed countries [17, 18].

Phage therapy has been used to control *Salmonella* in chickens with varying degrees of success. Sklar et al. used phages in a broiler chick model to demonstrate that *Salmonella* colonization of the cecum could be significantly reduced by almost 1 log_10_ Colony Forming Units (CFU)/g gut contents over 14-days by administering a cocktail of four phages in feed (10^9^ Plaque Forming Units (PFU)/g) [19]. Additionally, phage treatment appeared to reduce secondary infection signs in the birds as only three out of 10 animals in the phage-treated group showed mild inflammation on the air sacs while 8 out of 10 birds in the untreated control group showed signs of airsacculitis. Fiorentin et al. demonstrated that a single oral dose of three phages each at 10^11^ PFU could reduce *S*. Enteritidis colonisation of broiler chickens by 3.5 log_10_ CFU/g in the broiler chicken caecum when exposed to 7-days-old seeder chicks infected with 10^8^ CFU of *S*. Enteritidis [20].

Atterbury et al. [21] selected three lytic phage (isolated from poultry farms and wastewater in the UK) with a broad host range against *S.* Enteritidis, *S.* Hadar and *S.* Typhimurium. A 9.0 log_10_ PFU suspension of each phage was used to treat 36-day-old Ross broiler chickens which had been separately infected with the three different serotypes. All of the phages tested reduced *Salmonella* colonisation of the ceca, although only *S.* Enteritidis and *S*. Typhimurium were reduced significantly; by approximately 2.19–2.52 log_10_ CFU/g compared with the controls. Bacteriophage-insensitive mutants (BIMs) were recovered from phage-treated animals. However, this phage-resistant phenotype was not maintained in vitro following successive subculturing, nor in vivo when BIMs were introduced into a new group of birds in the absence of phage selective pressure.

Lim et al. demonstrated how phage could be used to prevent horizontal infection by *Salmonella* Enteritidis in a commercial layer chick seeder model [22]. Groups of 1-day-old chicks were challenged with 5 × 10^11^ CFU of *Salmonella* Enteritidis and for the next 21 days cohabitated with uninfected contact chicks while being treated in three independent groups with one of three titres (10^5^,10^7^ or 10^9^ PFU/g) of bacteriophage prepared as a feed additive. All the treatments significantly (*P* < 0.05) reduced the intestinal colonization by up to 1 log_10_ CFU/g at the end of the 21 days, with 70% of the contact chickens treated with the highest phage titre having no detectable *Salmonella* Enteritidis colonization. Borie et al. [23] administered a combination of three *Salmonell*a-specific phages (10^8^ PFU/mL/dose) via coarse spray or drinking water on 10-day-old chicks 24 h before the experimental challenge with 9.6 × 10^5^ CFU *S.* Enteritidis. Phage delivery both by coarse spray and drinking water significantly reduced the mean intestinal *S.* Enteritidis counts by up to 1.6 log_10_ CFU/mL .

Ahmadi et al. [24] determined the ability of phages to reduce *S*. Enteritidis in the ceca of Japanese quails. Phage ‘PSE’ was administered to groups of 8-day-old quail by oral gavage either prophylactically (10^5^ PFU) three days before challenge with 10^8^ CFU *S*. Enteritidis; or therapeutically immediately after *S*. Enteritidis challenge. Prophylactic administration reduced the detection of *Salmonella* in the ceca to 33.3 and 20%, 24 h and 7 days after PSE administration, respectively, while in the infected control group all birds tested positive for *S.* Enteritidis in the cecal tonsils. No such reduction was recorded in the birds treated therapeutically. In a further experiment, groups of 1-day-old quail were treated with 10^8^ PFU of phage PSE daily for six days, either by oral gavage or vent lip. On day four, these birds were challenged with 10^8^ CFU of *S*. Enteritidis. *Salmonella* was detected in the ceca of birds treated orally with phage PSE 6 h following *Salmonella* challenge, but not subsequently up to 35 days post challenge. Some birds (up to 2 out of 8) treated with phage PSE via the vent lip periodically tested positive for *S*. Enteritidis throughout the study period, but the majority remained free of *S*. Enteritidis.

Besides the application in poultry, the effect of phage cocktails against *Salmonella* in pigs has also been demonstrated. Wall et al. [25] administered 5 × 10^9^ CFU of *S*. Typhimurium γ4232 and, simultaneously, microencapsulated alginate beads containing 5 × 10^9^ PFU of 16 phages mixed as cocktail, to 3 to 4 week old pigs by gavage. The authors reported a 2 to 3 log_10_ CFU/g reduction in the *Salmonella* counts in the ileum, caecum and tonsils. In a different experimental design, market-weight pigs were challenged with 5 × 10^9^ CFU *S*. Typhimurium orally and then treated with 10^10^ PFU of microencapsulated phage cocktail 48 h later (administered orally three times, with 2 h interval between doses) followed by comingling with *Salmonella*-infected seeder pigs. *Salmonella* average cecal counts in the phage-treated pigs were significantly reduced by 1.4 log_10_ CFU/mL compared with untreated controls.

Saez et al. [26] administered a bacteriophage cocktail as a microencapsulated feed additive and demonstrated it can be an effective and practical way of reducing *Salmonella* colonization and shedding in pigs. The experimental design comprised 21 pigs randomly separated into three groups. Group 1 was given a feed additive containing a microencapsulated phage cocktail (5 × 10^11^ PFU per day) for 5 days before being orally challenged with 5 mL of 10^9^ CFU/mL *Salmonella* Typhimurium. Group 2 was given 60 mL of the phage cocktail (5 × 10^11^ PFU) every 2 h after the challenge, during a total of 6 h. Group 3 received no phage treatment, and all groups were orally challenged on the fifth day with 5 mL of 10^9^ CFU/mL *S*. Typhimurium bacterial suspension. The results showed lower faecal shedding of *S.* Typhimurium at 2 h post challenge (group 1 = 38.1%, group 2 = 71.4%, group 3 = 71.4%, *P* < 0.05) and 4 h post challenge (group 1 = 42.9%, group 2 = 81.1%, group 3 = 85.7%, *P* < 0.05) when the phage cocktail was administered as a feed additive. Additionally, *S*. Typhimurium counts in ileal and cecal contents were 1 log_10_ CFU/g lower in the phage-treated feed additive group compared with the control group.

Seo et al. [27] determined the therapeutic potential of a bacteriophage cocktail which was able to kill 34 reference strains and 99 isolates (107 tested) of *S*. Typhimurium. Groups of four-week-old pigs were given 5 mL of a phage cocktail at 10^9^ PFU/mL until the end of the study (15 days) and at day 7 were challenged with 10 mL of *S*. Typhimurium (ATCC140828) at 10^8^ CFU/mL. No *Salmonella* shedding was detected in the fecal samples 7 days post infection in the phage treated group compared with a mean colonisation of 1.0 log_10_ CFU/mL for the untreated control group.

The administration of *Salmonella* phages orally exposes them to potentially hostile conditions, such as low pH of the stomach/gizzard and the activities of bile and enzymes in the duodenum, which may impact bacteriophage viability. Various approaches have been used to mitigate against the potential damage which these conditions may cause, including concomitant administration of antacid [21], microencapsulation with chitosan/alginate [28] microencapsulation with alginate [29], microencapsulation with antacid/alginate [30] and liposome nanoencapsulation [31].

### Colibacillosis

Pathogenic strains of *Escherichia coli* in poultry are the causal agent of colibacillosis which is responsible for considerable mortality in poultry. *E. coli* colonization of the avian respiratory tract may progress down to the air sacs causing septicaemia and ultimately death [32]. In broiler chickens, Huff et al. [33] demonstrated the efficacy of administering a high titre mixture of phage by spray to reduce colibacillosis-associated mortality. In this model, high titres of two phage (SPR02, 2.6 × 10^8^ PFU/mL and DAF6, 2.35 × 10^9^ PFU/mL) were administered by spray at day 7, followed by challenge with a pathogenic *E. coli* injected directly into the thoracic air sac (5.6 × 10^4^ CFU) at day 7, 8 or 10. Phage treatment resulted in a significant reduction in mortality which was most pronounced when the phage were administered at the same time as the bacterial challenge (30% mortality vs 60% mortality for untreated control birds). Noteworthy, one experimental group of birds was found to be already infected with *E. coli* which was susceptible to lysis by phage SPR02. The authors suspected that this was due to a naturally-occurring infection, though this was not confirmed. These birds had a slight lower hatch weight, and a significantly higher mortality in the untreated control group (20%) and buffer spray control group (27%) compared with the phage treated group (3%). This suggested that phage administration might have provided therapeutic treatment for the pre-existing colibacillosis.

Huff et al. [34] again used phage SPR02 in a different phage therapy model. Groups of ten 3-day-old chicks were challenged with 10^3^ to 10^4^ CFU of *E. coli* by direct injection into the air sacs. One group was administered phage (10^3^ or 10^6^ PFU) simultaneously with the *E. coli*, the second group was administered phage via drinking water. Administration of phage via drinking water had no protective effect, whereas simultaneous injection was associated with a reduced mortality (25% or 5% for birds treated with 10^3^ and 10^6^ PFU respectively). This compared favourably with 80% mortality recorded for the untreated control group. However, the mixing of phage and host during administration is likely to produce artificially positive results, as the phage will have the opportunity to infect and replicate within the bacteria before they have a chance to establish an infection; effectively reducing the challenge.

Huff et al. [35] again used phages SPR02 and DAF6 to treat colibacillosis, this time by aerosol spray or intramuscular injection. The challenge used 5.96 × 10^4^ CFU of *E. coli* injected in the left thoracic air sac of 7-day-old chicks. The phage treatment using the aerosol spray (7.65 × 10^8^ and 2.83 × 10^9^ PFU/mL, DAF6 and SPR02, respectively) provided significant protection to the birds as shown by the low mortality of the treated group (20%) when compared to the control group (50%). However, if phage treatment was delayed by 24 or 48 h after bacterial challenge, no therapeutic benefit was observed. Conversely, birds treated with a combination of phages (1.88 × 10^9^ and 6.35 × 10^8^ PFU/mL of DAF6 and SPR02, respectively) by intramuscular injection had a significantly lower mortality (≤ 20%) compared with the control group (53%) whether phage were administered immediately or up to 48 h after bacterial challenge. These results reinforce the notion that the administration route plays a fundamental part in the phage therapy outcome, as the best results appear to be achieved by injecting phage into the birds, which given the nature of poultry farming in unlikely to offer a practical solution to colibacillosis.

Huff et al. [36] evaluated the potential synergy of antibiotics and phage treatment for colibacillosis. Groups of ten seven-days-old chicks were challenged with 6 × 10^4^ CFU of *E. coli* injected into the left thoracic air sac. This was followed immediately by injection of one of two phages directly into the thigh muscle (3.7 × 10^9^ and 9.3 × 10^9^ PFU per mL of phages DAF6 or SPR02, respectively). Enrofloxacin was introduced into the birds’ drinking water at 50 ppm for 7 consecutive days, starting immediately after *E. coli* challenge. High mortality (68%) was recorded for the untreated control group. This compared with 15% mortality in the phage-treated group, 3% mortality in the enrofloxacin-treated group and 0% for birds treated with both phage and antibiotic. This led the authors to suggests the occurrence of synergy between both therapeutic agents when used in combination with improved efficacy.

Oliveira et al. [37] tested phage delivery using a fine drop scatter spray in experimentally and naturally infected chickens. Groups of twelve 10-week-old Rhode Island Red chickens were challenged with 1 × 10^8^ CFU of avian pathogenic *E. coli* H839E, by injection in the left thoracic air sac. The phage-treated group was given a suspension of 1.5 × 10^9^ PFU phi F78E, orally and by spray. The results showed a significantly (*P* < 0.05) lower pathology score (≈2.5), morbidity (≈60%) and mortality (≈45%) in the phage-treated group when compared to the untreated control pathology (≈4), morbidity (≈100%) and mortality (≈75%) scores. Moreover, the average mortality was 25.0% lower, the average morbidity 41.7% lower, and the lesions found in carcasses were also less severe in the phage-treated group, when compared with the untreated controls.

El-Gohary et al. [38] tested the spray delivery of phages onto litter as a means to reduce colibacillosis. The surface of the litter in 3.9 m^2^ pens was sprayed with 200 mL of a 2.8 × 10^8^ CFU/mL of a culture of pathogenic *E. coli.* For phage-treated groups, the pens were immediately sprayed with 200 mL of a 8 × 10^8^ PFU/mL suspension of phage SPR02. The mortality of the control and the phage-treated groups was 25 and 5%, respectively. The authors suggest that sanitising the environment with phages reduces the level of the target pathogen to below the infectious dose, hindering the onset of bacterial diseases, and claim it is a practical and efficacious way to prevent colibacillosis in broiler chickens. However, the metabolic state of in vitro cultured bacteria, such as those sprayed on litter, may differ substantially from sub-lethally injured cells which may be found naturally in the farm environment. As such, it may be difficult to repeat these results in a real poultry farm scenario.

Besides respiratory infections in poultry, colibacillosis in a meningitis and septicaemia model were addressed in poultry with a phage therapy approach [39]. The experimental design used 3-week-old Rhode Island Red chickens infected with *E. coli* H247 K1+ via intramuscular or intracranial administration, followed by an intramuscular injection of phage R at 10^4^ or 10^6^ PFU. Mortality in the phage treated groups was zero, compared with 100% in the untreated control group. None of the birds treated with phage exhibited clinical signs of infection. Moreover, delaying phage treatment until clinical signs of disease were evident still led to considerable protection as all 10 untreated control birds died compared with 3 out of 10 for the phage-treated group. Prophylactic administration of phage up to two days before bacterial challenge was also effective in reducing mortality to 1 out of 9 in the phage treated group compared with 4 out of 9 in the control group. Phage R was able to multiply in the blood and penetrate the blood-brain barrier. Together, this data supports the idea that even acute infections might be amenable to phage treatment.

Pioneering work in phage therapy addressing colibacillosis in pigs (and also mice, cattle and sheep) was performed in the 1980s by Smith and Huggins [40–42]. In one study, diarrhoea was induced in piglets by giving them 3 × 10^8^ CFU of pathogenic *E. coli* O20:K101 987P to seven piglets and 13-16 h later they were treated by oral administration of a mixture (10^10^ PFU) of two phages (P433/1 and P433/2) or a single phage (P433/1). Symptoms of disease in phage-treated piglets ceased 18-22 h later, while the challenged untreated piglets were severely ill, markedly dehydrated, ataxic, mentally confused and if they had not been fed by stomach tube, the authors claim the whole group of seven piglets would have died [41].

Jamalludeen et al. [43] showed a beneficial effect of phages on weaned pigs infected with an enterotoxigenic *E. coli* O149:H10:F4. The pigs were inoculated orally by syringe with 10^10^ CFU of *E. coli*, followed by treatment with six phages (GJ1-GJ7) either individually or combined in a dose of 10^9^ PFU of each phage. These phages were administered either prophylactically (15 min after challenge) or therapeutically (24 h after challenge). The antibiotic florfenicol was used prior to the bacterial challenge in an attempt to enhance *E. coli* colonization. The prophylactic use of the six phages individually significantly reduced the duration and severity of the diarrhoea as shown by the clinical symptom score of < 4 when compared with ≈10 from the challenged control. Moreover, the therapeutic administration of a two phage cocktail significantly reduced the symptoms, the development of diarrhoea and the shedding of the pathogenic *E. coli* without changes to the commensal *E.coli* numbers [43]. The usage of bacteriophages as an in-feed additive for pigs, administered prophylactically, was regarded as safe as it had no adverse immunological effects, and may also result in improved weight gain [44–47].

### Campylobacteriosis

*Campylobacter* spp. is the most significant cause of acute bacterial food borne disease in the EU [48]. Approximately 95% of all reported cases result from infection with one species, *C. jejuni*. *Campylobacter* is highly adapted to the colonisation of the avian gut and has a relatively low infectious dose for humans (thought to be approximately 500 cells [49]). There is a host immune response, manifest by the sIgA antibodies titre, however it has little to no effect on the level of colonization of *C. jejuni* in broilers [50]. High counts of *Campylobacter* bacteria on broiler caeca may result in carcass contamination at the abattoir. It has been calculated that a reduction of *Campylobacter* counts on carcasses by 2 log_10_ may result in a 30-fold decrease in human campylobacteriosis [51]. The antibiotic resistance profile of 486 campylobacters isolated from retail chickens by the UK Food Standards Agency from 2016 to 17 found resistance to ciprofloxacin (251), tetracycline (322), nalidixic acid (247), streptomycin (18) and erythromycin (2). Multidrug resistance to three or more antibiotics was recorded for 17 isolates [52]. These results stress the need for an effective solution to deal with contamination of poultry carcasses with *Campylobacter*.

Wagenaar et al. [53] determined whether a phage preparation administered by oral gavage (from day 7 to 16) could protect 10-day-old Ross broiler chicks or adult chickens from a challenge with *C. jejuni* (10^5^ CFU/mL on day 10). The phage preparation did not show any protective effect in the birds, however, when administered after the bacterial challenge, a 3 log_10_ CFU/g reduction in the counts of *C. jejuni* was observed in the caeca of phage-treated birds. Loc-Carrillo et al. [54] selected two phages (CP8 and CP34) from a panel of 53 isolated from chicken faeces to use as candidates to reduce *Campylobacter* in chickens. The phages were selected on the basis of favourable in vitro replication kinetics and a broad host range. Ross broiler chickens were experimentally infected with *C. jejuni* isolates HPC5 and GIIC8 at various doses (from 2.7 to 7.8 log_10_ CFU) by oral gavage at 18 to 20 days of age. A single phage treatment (5–9 log_10_ PFU) was administered at 25 days of age by oral gavage. *C. jejuni* counts in the upper intestine and ceca of phage-treated birds were reduced by between 0.5 and 5 log_10_ CFU/g when phages were applied at ≥10^7^ PFU. Phage-resistant *C. jejuni* isolates were recovered from phage-treated birds (4%) but this was markedly lower than recovery of resistant isolates from in vitro studies (11%). The authors suggested that, in the absence of phage selective pressure, phage-resistant mutants may colonise the chicken gut less effectively. This interpretation is supported by the authors’ observation that when phage-resistant isolates are used to challenge birds in the absence of phage, 97% of campylobacters reverted to a phage-sensitive phenotype [54]. In a previous study, the same group showed that in 90 UK broiler flocks, counts of *C. jejuni* in the presence of naturally occurring bacteriophages were lower when compared to samples where phage could not be detected (5.1 vs 6.9 log_10_ CFU/g respectively) [55].

Lytic phages that infect *Campylobacter* have been categorized into three groups (I to III) based on the structure, genome size and receptor used to infect the host [56]; and phages from group II and II apparently use multiple host cell receptors for binding [57–59]. El-Shibiny et al. [60] recorded a 2 log_10_ CFU/g reduction in caecal counts of *Campylobacter* HPC5 48 h after administering a single 10^7^ PFU dose of group II bacteriophage CP220. The incidence of phage resistance in colonized chickens after phage treatment was shown to be residual, only around 2% of the population [60]. More recently, Hammerl et al. [61] used a combined treatment of group II and III phages. Groups of 20-day-old male Vrolix chicks were inoculated with 10^9^ CFU of *C*. *jejuni*. After 7 days the infected birds were administered a phage suspension of 5 × 10^8^ PFU of CP14 (group III), CP81 (group III) or CP68 (group II) either alone or combined. At 31 days of age the experimental birds were euthanised and the *C. jejuni* counts in caeca revealed a 1 log_10_ CFU/g reduction in cecal colonisation when treated with CP14 alone, compared with the control group. The addition of CP81 to CP14 did not improve this reduction. However, a 3 log_10_ CFU/g reduction was recorded when treatment with CP14 was followed by CP68 the next day. The authors claim the different host receptors used by group II and III phages are the underlying reason for both the significant reduction in *Campylobacter* counts and also for the lower levels of resistant isolates obtained when using a mixture group II and III phages (3%) when compared to the single CP14 phage (4%) or two phages from the same group III (8%).

As *Campylobacter* colonizes the caeca in birds, and does not appear to be very invasive, phage are usually administered orally. Carvalho et al. [62] found that the administration of a cocktail of three phages to broiler chickens by gavage and feed reduced *C. jejuni* and *C. coli* colonisation in broiler chicken faeces by approximately 2 log_10_ CFU/g. The authors report the counts of *Campylobacter* were maintained 1 log_10_ CFU lower in the phage-treated group when compared to the untreated control. However, phage resistant isolates recovered from faeces (13%) did not show a reduced ability to colonize the chicken guts or revert to a phage-sensitive phenotype. More recently, the impact on the microbiota of broiler chickens infected with *Campyobacter jejuni* HPC5 after treatment with a two phage cocktail was determined [63]. The authors showed a 2 log_10_ CFU/g reduction on the *Campylobacter* counts in cecal content, that in vivo the phages replicate and maintained as a stable population and, additionally, the infection of *C. jejuni* by the phages tested did not affect the microbiota [63].

### Clostridiosis

*Clostridium perfringens* is the causal agent of necrotic enteritis, a disease that affects chickens and whose pathogenesis is incompletely understood. The involvement of toxins and hydrolases secreted by the bacterium are thought to be relevant for the virulence and intestinal colonization by the anaerobic *C. perfringens* [64]. Additionally, parasites of *Eimeria* species that colonize the small intestine, such as *Eimeria maxima* and *Eimeria acervulina*, are known to predispose to necrotic enteritis by leaking of plasma to the intestinal lumen which provides a necessary growth substrate for extensive proliferation of *Clostridium perfringens* [65]. Phage treatments have shown some efficacy in reducing the symptoms and disease progression in chickens. In a study using a total of 900 birds in various experimental designs, Miller et al. [66] showed that oral administration of a five phage cocktail at 10^5^ PFU/mL by oral gavage or drinking water to experimentally infected Cobb broiler chickens (0 to 42-days old) with *C. perfringens* resulted in a 92% reduction in mortality compared with the untreated control group. Additionally, the authors conclude that in the 0–42 days period, the specific cocktail used (INT-401) increased weight gain and feed conversion ratios in both the phage-in-water group (2.618 ± 0.059 kg) and phage-in-feed group (2.547 ± 0.059 kg) when compared to the challenged untreated group (2.296 ± 0.059 kg), and may be an effective therapy to control the necrotic enteritis caused by *C. perfringens*.

*C. perfringens* is a Gram-positive bacterium, this implies that the thick peptidoglycan layer is the outmost barrier exposed to the environment. The phage-encoded endolysins, enzymes that target and hydrolyse specific bonds within the peptidoglycan mesh, have been reported to suffice to achieve bacterial lysis [67]. The usage of purified endolysins from phages that target *C. perfringens* is shown as a promising route to reduce the colonization or treat the infection by this pathogen as has been described and subject of review elsewhere [8, 68–70].

## Conclusions

The emergence of antibiotic resistant zoonotic pathogens in the food chain is a growing public health problem worldwide. The lack of new antibiotics coming onto the market necessitates development of alternative strategies to deal with these bacteria. Bacteriophages were used in veterinary applications soon after their discovery more than a century ago. Whilst the efficacy of phage therapy varies according to the bacterial target and complexity and location of the infection site(s), most recent studies in intensively-reared livestock have found that these pathogens can be significantly reduced using phages. This may have a beneficial effect on both animal and human health, and in some instances may lead to greater productivity of the industry. Highly-integrated production systems, found in the poultry industry for example, are more amenable to phage therapy, as a single company can control all aspects of meat production before the point of retail sale. Potentially, this allows the flexibility to introduce phage at various points, from feed/water or sprays at the farm level to wash treatments and modified packaging at the abattoir. However, in the EU there is no regulatory framework which would allow such interventions. Bacteriophage do not fit easily into existing EU regulations regarding the use of food additives or food processing aids, which is a significant obstacle.

The emergence of phage-resistant bacterial pathogens is a threat analogous to the development of antibiotic resistance. However, resistance to one phage does not necessarily result in resistance to others, and there appears to be a fitness cost to resistance in the absence of phage, at least in some cases. These factors will be important when designing phage interventions in the future, which may comprise cocktails targeting several different receptors, thus minimising the probability of resistance emerging. In this context, pathogens which are more genetically homogeneous, such as *Staphylococcus aureus*, may be more attractive targets for phage therapy than genetically diverse hosts such as *E. coli,* as fewer phage will be needed to cover the range of clinical strains circulating in a population at any point in time. This may also influence the overall phage treatment strategy as phage deployed prophylactically, rather than therapeutically, against bacteria such as *E. coli* are less likely to result in success than for *Staphylococcus aureus*. Also, whilst entry of phage into the wider environment may be more controllable in intensively-reared livestock, some release is inevitable and may necessitate the regular reformulation or cycling of cocktails in order to circumvent resistance and maintain efficacy. Given the challenges of meeting the growing demand for meat over the next century, viable alternatives to antibiotics will be needed to control disease in increasingly intensifying production systems. However, like antibiotic chemotherapy and vaccination, this is unlikely to offer a panacea.

## Data Availability

Not applicable.

## Abbreviations

BIM: Bacteriophage-insensitive mutants
CFU: Colony Forming Units
EFSA: European Food Safety Authority
FAO: Food and Agricultural Organization
PFU: Plaque Forming Units
